# Editorial: The Relationships Between Infectious Agents and Dementia

**DOI:** 10.3389/fncel.2022.831374

**Published:** 2022-02-02

**Authors:** Lay Khoon Too, Nicholas H. Hunt, Noriko Shinjyo

**Affiliations:** ^1^Faculty of Medicine and Health, Save Sight Institute, The University of Sydney, Sydney, NSW, Australia; ^2^Discipline of Pathology, Faculty of Medicine and Health, The University of Sydney, Sydney, NSW, Australia; ^3^Laboratory of Immune Homeostasis, WPI Immunology Frontier Research Center, Osaka University, Osaka, Japan; ^4^School of Tropical Medicine and Global Health, Nagasaki University, Nagasaki, Japan

**Keywords:** neuroinflammation, synaptic dysfunction, infection, brain pathology, dementia

Many infectious agents are capable of disrupting the normal functioning of the central nervous system (CNS) by invading the brain parenchyma. Some of these pathogens, for example encephalitic viruses and meningitis bacteria, cause acute perturbations, whilst the effects of others, such as prions and *Toxoplasma gondii*, are realized much more slowly. The protozoan parasite *Plasmodium falciparum* can cause acute fatal cerebral complications without extravasating into brain tissue.

During the last few years, evidence has gradually accumulated suggesting that certain pathogens may interfere with the normal functioning of the brain, over extended periods, without entering the CNS. Likely mechanisms include releasing soluble factors that can cross the blood-brain barrier, or inducing their production by the host immune system, or both. It has been hypothesized that these sorts of mechanisms may contribute to the neurodegeneration that is central to dementias, including Alzheimer's disease.

This Special Issue of *Frontiers in Cellular Neuroscience—Cellular Neuropathology* brings together several review and original research articles that explore these topics. The authors have evaluated from different standpoints the etiology of neuroinflammation, whether acute or chronic, and have critically examined the evidence concerning the mechanisms through which CNS or peripheral infection may relate to the development of dementia in some cases.

Bacterial meningitis is an inflammation of the meninges, the membranous coverings of the CNS. Mostly caused by blood-borne bacteria, such as *Streptococcus pneumoniae, Neisseria meningitidis*, and *Haemophilus influenzae*, bacterial meningitis in general is an acute condition with a high fatality rate. However, it also can cause long-lasting neurological sequelae in survivors. Two mini-review articles address the link between bacterial meningitis and neurodegenerative disorders from different perspectives, each providing original insights on the mechanisms through which blood-borne bacteria may cause neurodegeneration. Neuroinflammation is identified as an important common mechanism. Potential therapeutic approaches and future research directions are also suggested.

In their mini-review, Farmen et al. describe the molecular mechanisms by which bacterial pathogens can enter the brain parenchyma and cause neurodegeneration. Several molecular mechanisms potentially leading to neuronal cell death are explained, including direct neurotoxic effects of bacterial products and indirect routes mediated by microglial activation and neuroinflammation. They suggest therapeutic approaches through targeting host molecules that are involved in bacterial entry into the brain, by enhancing neuroprotection, and also through modulating immune responses. The authors point out the importance of identifying virulence factors responsible for the processes leading to neuronal loss. They conclude that, whilst treatments targeting neuroinflammation may prove useful for preventing neurodegeneration, more epidemiological studies are needed to understand the molecular mechanisms behind the short-term and long-term impacts of bacterial infection on cognitive ability, in order to effectively prevent neurological sequelae.

Alzheimer's disease and age-related macular degeneration are neurodegenerative disorders with distinct pathologies, but the two diseases share a number of common risk factors. Whilst an infectious etiology for Alzheimer's disease has gained considerable recognition in recent years, any link between infection and age-related macular degeneration is less recognized. By providing comparative reviews of Alzheimer's disease and age-related macular degeneration, Too et al. suggest common mechanistic pathways at the cellular and molecular levels, particularly focused on neuroinflammation. They identify several immune components as key players in bacterial meningitis and the two neurodegenerative disorders, supporting their views on the link between bacterial meningitis and neurodegeneration. Further, they outline the current treatment options for bacterial meningitis and discuss their potential for the therapy and prevention of Alzheimer's disease/age-related macular degeneration. They also suggest possible alternative scenarios for the infectious etiology of neurodegeneration and point out unresolved issues that must be addressed for the development of safe and effective therapeutic strategies.

Complement system activation is a keystone of the host anti-microbial response. Bacterial meningitis is a prominent example of the importance of this system for eliminating pathogens within the CNS. However, the neuroinflammatory response to the presence of the pathogens is a major cause of neuronal damage and dysfunction, which may be fatal or have deleterious long-term impacts on cognitive performance in survivors. A wide range of other pathogens—viruses, fungi and parasites—also activate the complement system within the CNS. Shinjyo et al. provide a comprehensive overview and evaluation of the significance for the development of dementia and other brain disorders of pathogen-induced complement activation within the CNS. Complement proteins are synthesized within the brain and have numerous non-immune roles, for example regulating some aspects of neuronal development and responses to injury. The authors point out that both over- and under-expression, or activation, of some components of the complement cascade are associated with brain disorders in experimental models and human disease. They also consider the association between complement activation and prion diseases. The authors bring together numerous strands of evidence concerning the potentially beneficial and deleterious effects of complement activation in the CNS response to pathogens. Finally, they consider the possible significance of these processes in the insidious development of neurodegeneration and dementia, pointing out that the outcomes of complement system activation in the CNS may be context-dependent and influenced by numerous regulatory factors.

Turning now to viruses, Hosseini et al. study the important concept that infection with common respiratory viruses might promote Alzheimer's disease through inducing microglial activation and, thereby, neuroinflammation. They report on work that builds on their previous finding that non-neurotropic influenza virus infection leads to prolonged cognitive impairment in mice. Microglial activation, neuroinflammation and the loss or functional impairment of neurons were suggested to mediate those effects. They now have gone on to show that sub-lethal inocula of a non-neurotropic strain of influenza virus exacerbated the progressive CNS changes occurring in a transgenic mouse model of Alzheimer disease. Among the neuropathological features that were studied, microglial activation, amyloid-β plaque load, onset of synapse loss and decreased synaptic plasticity were all more prominent in the virus-infected animals than in their sham-infected counterparts. These factors likely explain the greater degree of cognitive impairment in the transgenic mice infected with the non-neurotrophic influenza virus.

Several historical lines of evidence have supported a link between infectious agents and the development of neurodegenerative diseases, especially Alzheimer's disease. Taking advantage of advances in high-throughput research technologies in the last few decades, multiple research groups have progressively explored the composition of the microbial fauna within the human body system that is associated with health and disease. Given that the gut and oral cavity are two important homes for the large diversity of microbiota, Narengaowa et al. reviewed the bi- and tri-directional oral-gut-brain interactions that might underlie the pathogenesis of Alzheimer's disease. By evaluating evidence from various sources, the authors postulate links between the oral/gut microbiota and the incidence of Alzheimer's disease. These might be the consequence of microbial pathogens entering the CNS through the breach of a protective barrier during a disease, e.g., gingivitis, or via microbial secretions that permeabilize the blood-brain barrier. In either case, the neuropathologies of Alzheimer's disease could be triggered. Alternatively, the oral/gut microbiota may indirectly lead to the development of Alzheimer's disease through the release into the bloodstream of neuroactive agents that subsequently enter the CNS, initiating a cascade of neuroinflammatory processes.

Growing evidence suggests an association of metabolic disorders with neurodegenerative maladies, such as Alzheimer's disease. In general, energy metabolism and the immune system are tightly related. Shinjyo and Kita discuss in their review how interactions between infectious agents and metabolism in the brain and periphery might contribute to neurodegeneration. Specifically, two metabolic hormones, insulin and leptin, that are key players both in energy homeostasis and neurocognitive development are comprehensively explored. The review brings together numerous lines of evidence that potentially link two factors: first, the bidirectional relationship between the sustained inflammation and metabolic disturbance caused by pathogens and, second, chronic inflammation and increased risk of neurodegenerative disorders. The authors go on to analyze evidence that supports a possible contribution of dysregulated leptin/insulin signaling pathways to neurological disorders caused by various central and peripheral infectious agents. These include West Nile virus, *Porphyromonas gingivalis*, human immunodeficiency virus, Borna disease virus, and Canine distemper virus. They conclude their review by identifying key questions that remain to be answered before any therapeutic benefits of targeting immunometabolic pathways might be harnessed.

The articles in this Special Issue highlight the pivotal role of neuroinflammation in disrupting the normal operations of the CNS across a spectrum of different initiating agents. In the case of some viral and bacterial pathogens that invade and replicate within the intracranial space, the inflammatory response is acute, brutal, and makes a major contribution to neuronal damage. In contrast, other pathogens may perturb brain function through systemic actions that induce insidious neuroinflammatory processes which smolder over prolonged timeframes. Classic inflammatory mediators such as complement proteins and immune mediators seem to be implicated in both the acute and chronic neuroinflammatory etiologies. Anti-microbial agents are established front-line treatments for neuroinvasive pathogens, but complementary therapeutics that dampen neuroinflammation might be beneficial, by lessening cellular damage, if they do not interfere with the host immune response. The recent findings considered in the Special Issue suggest new approaches to preventing or slowing the onset of dementias by ameliorating the long-term effects of infectious diseases, but much more detail is needed about the key pathways that are involved.

## Author Contributions

LKT, NH, and NS wrote and critically reviewed the manuscript. All authors contributed to the article and approved the submitted version.

## Conflict of Interest

The authors declare that the research was conducted in the absence of any commercial or financial relationships that could be construed as a potential conflict of interest.

## Publisher's Note

All claims expressed in this article are solely those of the authors and do not necessarily represent those of their affiliated organizations, or those of the publisher, the editors and the reviewers. Any product that may be evaluated in this article, or claim that may be made by its manufacturer, is not guaranteed or endorsed by the publisher.

